# The independence of and associations among apoptosis, autophagy, and necrosis

**DOI:** 10.1038/s41392-018-0018-5

**Published:** 2018-07-01

**Authors:** Qi Chen, Jian Kang, Caiyun Fu

**Affiliations:** 1 College of Life Sciences, Zhejiang Sci-Tech University, Hangzhou, 310018 China; 2 Zhejiang Provincial Key Laboratory of Silkworm Bioreactor and Biomedicine, Hangzhou, 310018 China; 3 Cancer Signalling Laboratory, Oncogenic Signalling and Growth Control Program, Peter MacCallum Cancer Centre, 305 Grattan street, Melbourne, VIC 3000 Australia; 4 Department of Pharmaceutical Chemistry and the Cardiovascular Research Institute, University of California San Francisco, 555 Mission Bay Blvd. South, San Francisco, CA 94158 USA; 5 Key Laboratory of Tumor Molecular Diagnosis and Individualized Medicine of Zhejiang Province, Hangzhou, 310014 China

## Abstract

Cell death is an essential biological process for physiological growth and development. Three classical forms of cell death—apoptosis, autophagy, and necrosis—display distinct morphological features by activating specific signaling pathways. With recent research advances, we have started to appreciate that these cell death processes can cross-talk through interconnecting, even overlapping, signaling pathways, and the final cell fate is the result of the interplay of different cell death programs. This review provides an insight into the independence of and associations among these three types of cell death and explores the significance of cell death under the specific conditions of human diseases, particularly neurodegenerative diseases and cancer.

## Targeting the interconnections of cell death to treat disease

New understandings of the interconnectivity of cell death pathways could provide novel therapeutic targets for cancer and neurodegenerative diseases like Alzheimer’s. Caiyun Fu of China’s Zhejiang Sci-Tech University and colleagues reviewed the latest research into the mechanisms and connections between the different types of cell death. Apoptosis, ‘programmed’ cell death; necrosis, ‘accidental’ cell death; and autophagy, the means by which cells destroy damaged internal organelles, each has independent molecular mechanisms that can also be simultaneously activated, operating in parallel in response to stress. Targeting these interconnected mechanisms with drugs could treat neurodegenerative diseases like Alzheimer’s and Parkinson’s, in which deregulated cell death plays a major role, and cancer, in which highly proliferating cells escape death pathways.

## Introduction

For unicellular organisms, cell death is the end of life. However, for multicellular organisms, cell death is an essential biological process for physiological growth and development. Deregulation of cell death is involved in the pathogenesis of a wide range of human diseases, such as neurodegenerative diseases and cancer^1^. Three classical forms of cell death—apoptosis, autophagy, and necrosis—display distinct morphological features by activating specific signaling pathways^2^. In brief, apoptosis is a caspase-mediated programmed cell death^3,4^ that is characterized by chromosome condensation, nuclear fragmentation, and membrane blebbing^5^. In contrast to apoptosis, necrosis is considered to be an unregulated, accidental cell death caused by nonspecific, or non-physiological stress inducers and is characterized by the expansion of cellular organelles, plasma membrane rupture, and subsequent inflammatory responses caused by release of the intracellular contents^6,7^. The third form of cell death, autophagy, is accompanied by the formation of the autophagosome, which is a bilayer vesicle containing damaged organelles, proteins, and other cytoplasmic components. The autophagosomes fuse with the lysosomes, degrading cellular macromolecules and organelles and producing renewable energy and metabolites for cells^8^. Autophagy acts as a pro-survival mechanism but can also induce autophagic cell death, which is currently an active research area in cell death^9,10^. Our understanding has been rapidly expanded in the last decades owing to the great advances in cell death research. Identification of the programmed forms of necrosis^11^ has changed our perception about necrosis. More importantly, we have started to appreciate that the molecular mechanisms of various types of cell death are distinct but also overlapping. There are multiple signaling pathways independently controlling different types of cell death. However, they are interconnected, can be activated simultaneously, and can operate in parallel in cells in response to stress.

**Fig. 1:**
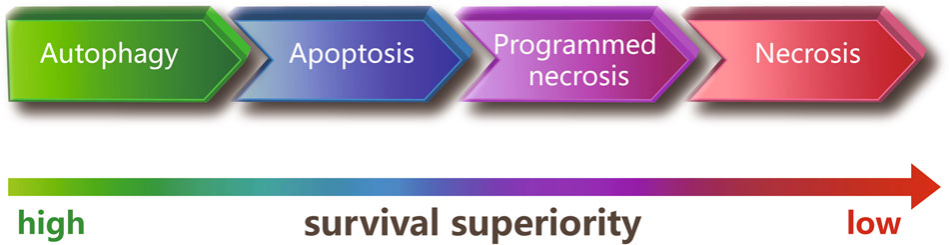
Survival superiority among the different types of cell death

Necrosis and apoptosis are two types of cell death with different mechanisms^5,12^. Autophagy can be described as a degradation mechanism rather than as a form of cell death, although it can also induce cell death^9^. Of the cell death types, autophagy has the highest survival superiority, followed by apoptosis, with necrosis having the lowest survival superiority. Autophagy is instinctively induced prior to apoptosis when cells are stimulated by stress, and apoptosis rather than necrosis is induced if autophagy is inhibited or ineffective^8,13–16^. Thus, two or three types of cell death may be induced simultaneously or successively when cells are exposed to certain stimuli. If the three types of cell death are placed on an axis according to their survival superiority, autophagy, and necrosis would be placed at opposing ends, whereas apoptosis would be placed in the middle; furthermore, programmed necrosis would be placed between necrosis and apoptosis (Fig. 1).

Herein, we review the different types of cell death, discuss the specific mechanisms involved in each type of cell death and connections among them, and explore the impact of different types of cell death on disease treatment.

## Distinct characteristics of apoptosis, necrosis, and autophagy

### Apoptosis

**Fig. 2:**
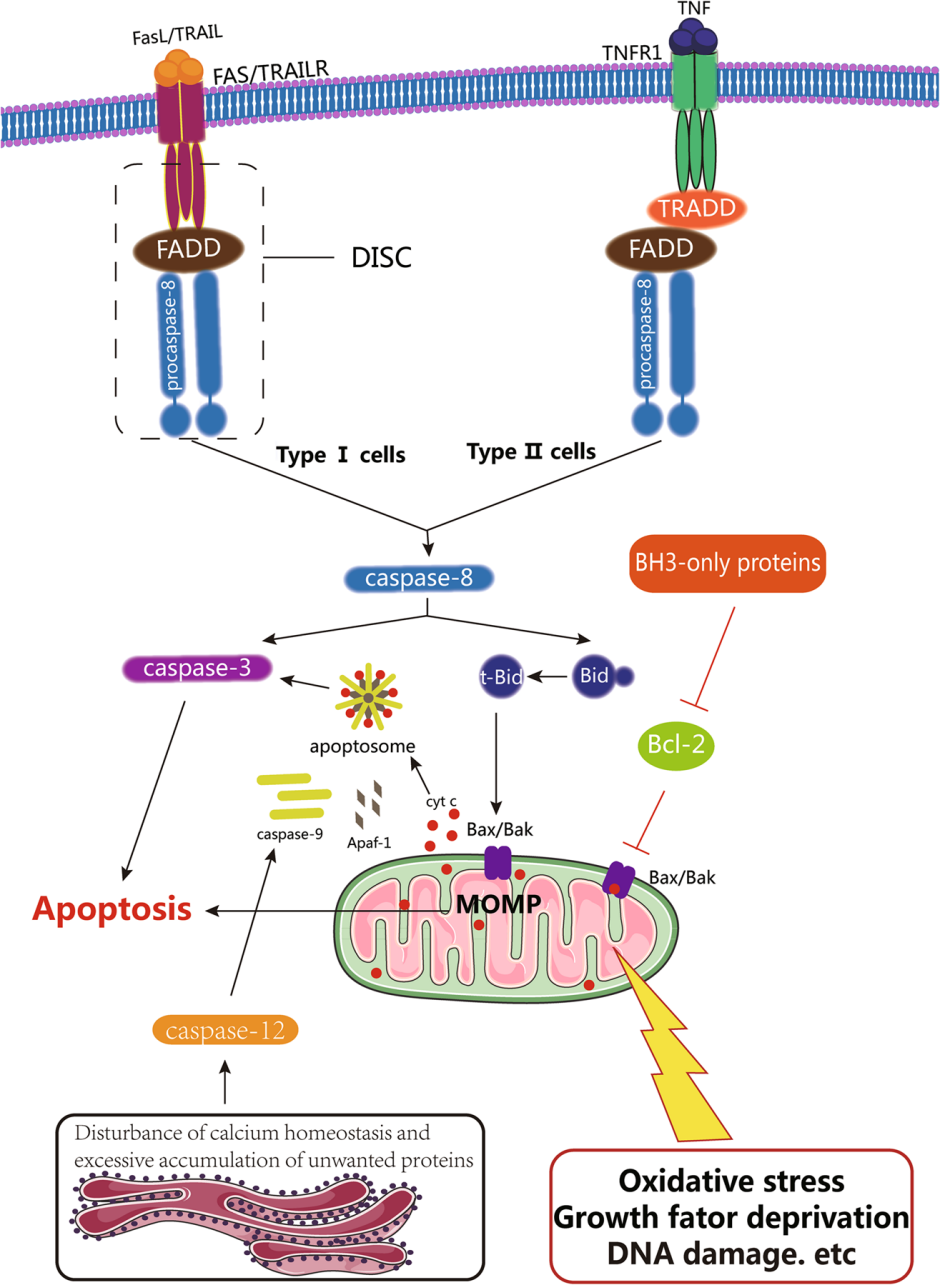
Mechanisms of apoptosis. In the exogenous pathway, the binding of FASL, TNF-α, or TRAIL to their corresponding receptors can transform procaspase-8 to caspase-8 through autohydrolysis. In type I cells, activated caspase-8 can activate caspase-3, followed by apoptosis. In type II cells, activated caspase-8 can hydrolyze Bid to tBid, and then tBid interacts with Bax/Bak, which is located on mitochondria, to induce apoptosis. In the intrinsic apoptosis pathway, DNA damage, growth factor withdrawal, oxidative stress, or toxic damage can destroy the homeostasis of the mitochondria, typically controlled by the Bcl-2 family members, and can lead to increased mitochondrial membrane permeability to induce cytochrome c release from the intermembrane space of the mitochondria. In addition, the released cytochrome c can interact with Apaf-1 and caspase-9 to activate caspase-3 and induce apoptosis. In the endoplasmic reticulum stress-induced apoptosis pathway, the disturbance in calcium homeostasis and excessive accumulation of unwanted proteins in the endoplasmic reticulum induce caspase-12-mediated apoptosis, in which activated caspase-12 translocates from the ER into the cytosol to directly cleave caspase-9 and then activate caspase-3

Apoptosis is generally considered a caspase-mediated programmed cell death^3,4,17,18^. Apoptotic cells display distinct morphological characteristics, including cell shrinkage, chromosome condensation, nuclear fragmentation (late stage), plasma membrane blebbing and the formation of apoptotic bodies, and exhibit biochemical changes, such as the exposure of phosphatidyl-l-serine on the outer plasma membrane (early stage)^19–21^. Apoptosis can be activated via either the death receptor-mediated apoptosis pathway, the mitochondria-dependent apoptosis pathway or endoplasmic reticulum (ER) stress-induced apoptosis pathways (Fig. 2)^22^.

The death receptor-mediated apoptosis pathway is activated upon the binding of the Fas ligand, TNF-α (tumor necrosis factor α), or TRAIL to the corresponding death receptors^23,24^. The adaptor protein FADD^23,25^ and the procaspase-8 protein form a complex, namely, death-inducing signaling complex (DISC). In DISC, procaspase-8 is activated by autohydrolysis^26^. The activated caspase-8 transduces the apoptosis signal through either the activation of caspase-3 or cleavage of Bid to truncated Bid (tBid). tBid translocates to the mitochondria, resulting in conformational changes in Bax and Bak and their oligomerization for pore formation in the outer mitochondrial membrane^26,27^.

The mitochondrial-dependent pathway can be activated by various stress inducers such as DNA damage, growth factor withdrawal, and oxidative stress^28,29^. The Bcl-2 family of proteins controls this intrinsic pathway by regulating the permeability of the mitochondrial outer membrane^30–32^. Upon release from the mitochondria into the cytoplasm, cytochrome c combines with Apaf-1 to promote caspase-9 activation, which, in turn, activates effector caspases^33,34^ to trigger a cascade of proteolytic events.

In addition, ER stresses, such as calcium homeostasis disturbance, excessive unfolded, or misfolded protein accumulation in the ER, nutrient deprivation, and hypoxia, can induce apoptosis. This apoptosis is mediated by caspase-12, an ER-resistant caspase^22^. Activated caspase-12 directly cleaves caspase-9 after translocation from the ER into the cytosol, followed by caspase-3 activation^35^. The molecular mechanisms of activation of caspase-12 during ER stress include forming a complex with the inositol-requiring enzyme-1α-TNF receptor-associated factor 2 (TRAF2) complex^36^, or by calpains, a family of Ca^2+^-dependent intracellular cysteine proteases^37^.

### Autophagy

**Fig. 3:**
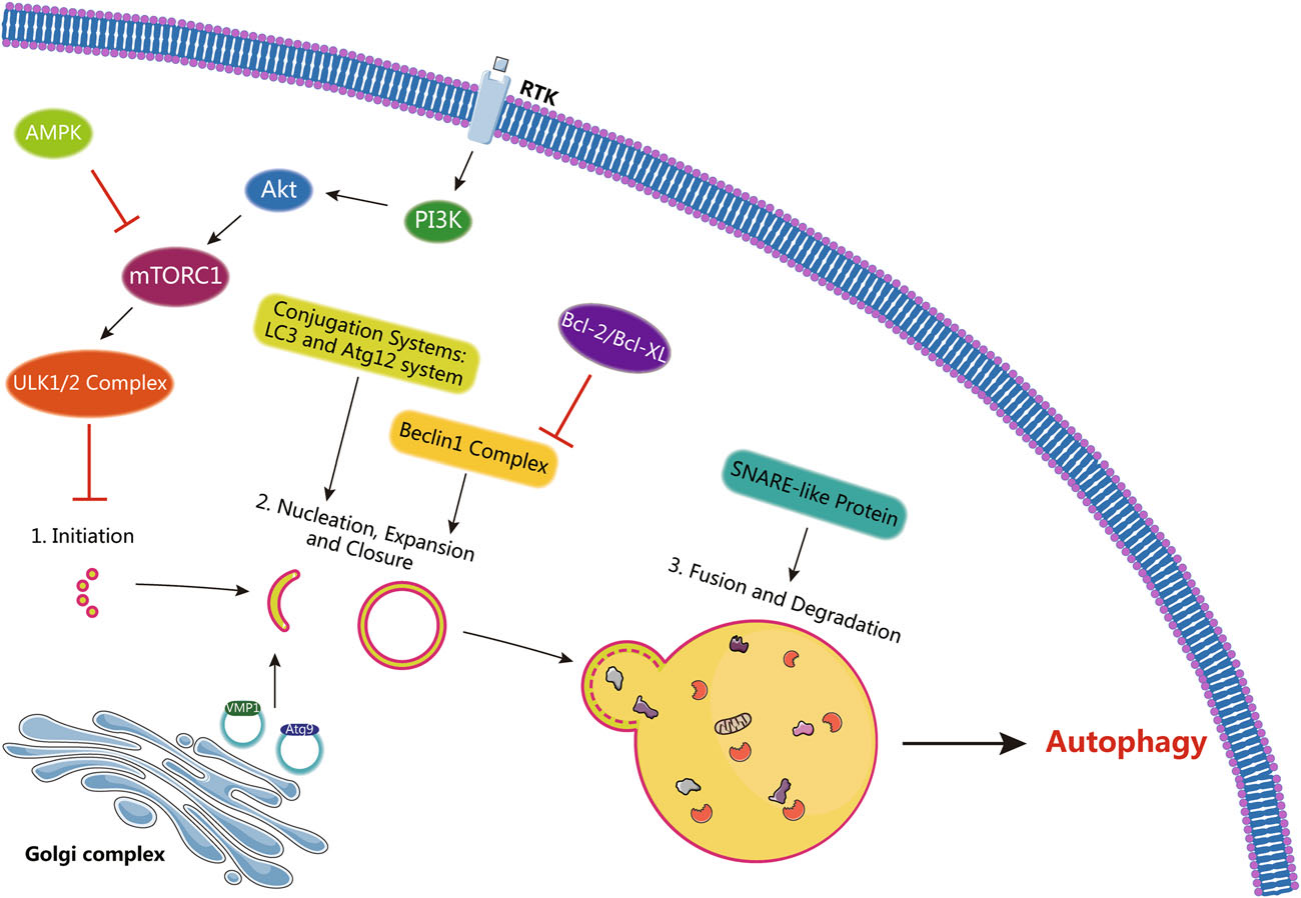
Mechanisms of autophagy. The mechanisms of autophagy can be divided into four steps, initiation, nucleation, expansion and closure, and fusion and degradation. In mammals, the assembly of the ULK1/2 complex is necessary for the formation of the phagophore assembly site, whereas the ULK1/2 complex is regulated by mTORC1, which is positively regulated by PI3K/AKT and negatively regulated by AMPK. Growth factors activate the PI3K/Akt pathway through receptor tyrosine kinases (RTKs). The Beclin1 complex, which is usually suppressed by Bcl-2, is activated and drives the isolation membrane to nucleation, and the transmembrane protein Atg9 and vesicle membrane protein VMP1 may be involved in the transport of lipids to the isolation membrane. In addition, two ubiquitin-like protein-conjugated systems (Atg12 and LC3 systems) are needed in this process. Subsequently, the autophagosome and lysosome fusion is mediated by SNARE-like protein, and, finally, various lysosomal enzymes hydrolyze all types of damaged organelles, proteins, lipids, and nucleic acids

Autophagy is a self-degradative process in response to various stresses, including nutrient deficiency, organelle damage, hypoxia, reactive oxygen species (ROS), ER stress, and drug treatment. The process of autophagy involves four key steps—initiation, nucleation, fusion of autophagosome and lysosome, and hydrolyzation. Our understanding of the molecular mechanisms of autophagy starts from research in yeast. A set of autophagy regulatory molecules was identified by genetic screening in yeast, particularly autophagy (Atg)-related proteins, which are the main players in autophagy. The assembly and aggregation of the Atg1 complex, which includes Atg1, Atg13, Atg17, Atg29, and Atg31^38,39^, are required for the formation of the phagophore at the initiation step^40^. However, in mammals, the UNC-51-like kinase 1 (ULK)-mAtg13-FIP200 complex, comprising the homologous analogs to yeast Atg1, Atg13, and Atg17, is formed^41^. At the step of nucleation, phagophore formation at the ER and other membranes is controlled by a complex of the class III PI-3 kinase VPS34, Atg6 (known as Beclin1 in mammals), Atg14, and Vps15. Atg9 and vesicle membrane protein VMP1, which circulate in the Golgi complex, autophagosomes, and endosomes, may be involved in the transport of lipids to the isolation membrane^42,43^. The expansion and closure of the autophagosome require two ubiquitin-like protein-conjugated systems, namely, Atg12 and Atg8 (Atg8 is also known as LC3 in mammals)^44^. The Atg12 system includes five Atg proteins, Atg5, Atg7, Atg10, Atg12, and Atg16^45–47^. Atg12 is activated by Atg7, which is an E1-like enzyme^45^, and is then transferred to the E2-like enzyme Atg10^47^. Finally, the C-terminal glycine of Atg12 covalently binds to the Lys149 side chain of Atg5 before binding to the dimer protein Atg16 to form the E3-like complex^45^. The Atg8 system including four Atg proteins, Atg3, Atg4, Atg7, and Atg8, represents another ubiquitin-like protein-conjugated system^48^. Atg8 is cleaved by Atg4, a cysteine protease, and exposes its C-terminal glycine residue (LC3 I in mammals)^49^. Atg8 is further activated by Atg7, an E1-like enzyme and is then transferred to Atg3, an E2-like enzyme^48^, before covalently binding to the amidogen of PE through the E3-like Atg12-Atg5-Atg16 complex^48,50^. The Atg8-PE covalent structure (LC3 II in mammals) confers Atg8 membrane tethering and hemifusion ability and plays a critical role in autophagosome formation. LC3 II is associated with both the outer and inner membranes of the autophagosome and is a typical marker of autophagy formation. The Atg8-PE covalent structure can be reversibly cleaved to Atg8 by Atg4 for the recycling of Atg8. Subsequently, the fusion of autophagosome and lysosome is mediated by SNARE (soluble N-ethylmaleimide-sensitive factor attachment protein receptor)-like proteins^51–54^. Finally, at a low pH, various lysosomal enzymes hydrolyze all types of damaged organelles, proteins, lipids, and nucleic acids^40,55^. A diagram of the autophagy process is shown in Fig. 3.

### Necrosis and necroptosis

Unlike apoptosis, necrosis is often considered to be an unregulated and accidental cell death^2^. However, the identification of programmed necrosis supported the existence of multiple nonapoptotic regulated cell death mechanisms. Several types of programmed necrosis have been reported, including necroptosis^56^, parthanatos^57^, ferroptosis^58^, pyroptosis^59^, and NETosis^60^. Here, we focus on necroptosis, a type of regulated necrotic cell death that shares several key signaling pathways with apoptosis. Most of the knowledge regarding necroptosis originated from investigations of TNF signaling. TNF is a pleiotropic cytokine that plays an important role in the process of inflammation^61^. TNF is also a potent cell death inducer under certain conditions through binding to TNFR^12^. Although an early study revealed that TNF-induced RIPK1-mediated caspase-independent cell death^62^, TNF-induced nonapoptotic cell death did not attract much attention, until researchers further uncovered that cells executed necrosis-like death when apoptosis was blocked^63,64^.

Necroptosis is initiated by the engagement of death receptors, such as TNFR1^65–67^ and Toll-like receptors (TLRs)^68–70^. TNF binding to the death receptor TNFR induces the conformational changes of TNFR, which recruits multiple proteins, including TNFR1-associated death domain protein (TRADD), RIPK1, TRAF2, E3 ubiquitin ligases, cIAP1/2, and LUBAC, to form TRADD and the RIPK1-dependent complex I. This multi-protein complex transduces pro-inflammatory and pro-survival signals by recruiting TGF-activated kinase 1 (TAK1)-binding protein (TAB) complexes and Iκb kinase (IKK) complexes consisting of IKK1, IKK2, and NF-κB essential modulator (NEMO)^71,72^ to activate NF-κB signaling, AP-1 signaling, and mitogen-activated protein kinase signaling. When RIPK1 is deubiquitinated by cylindromatosis lysine 63 deubiquitinase^73^, complex I becomes unstable and renders the dissociation of RIPK1 and the formation of another complex, termed complex IIa, by interacting with TRADD, FADD, pro-caspase-8, and FLIP^73^. Pro-caspase-8, together with FLIP_L_, cleaves RIPK1 to prevent necroptosis and activate apoptosis signaling^16,74,75^. TNF–TNFR signaling can also induce apoptosis through the formation of complex IIb when the function of IAP (inhibitors of apoptosis)^16^, TAK1^76^, NEMO, and/or Pellino3 is blocked^77^. This complex IIb comprises RIPK1, RIPK3, FADD, pro-caspase-8, and FLIP_L_ and causes RIPK1-dependent apoptosis^15^. However, complex IIb may be further transformed into the necrosome, a microfilament-like complex, when the levels of RIPK3 and mixed lineage kinase domain-like (MLKL) are sufficiently high and the activity of caspase-8 is inhibited^78^. Oligomerization and phosphorylation of RIPK3 in the necrosome lead to the recruitment and phosphorylation of MLKL^78,79^, and then MLKL translocates to the plasma membrane and causes membrane damage and necroptosis^80^. In addition, the necrosome interacts with mitochondrial serine/threonine protein phosphatase PGAM family member 5 on the mitochondrial membrane and activates mitochondrial fission factor dynamin-related protein 1 to induce necroptosis through mitochondrial fragmentation^81^. As mentioned above, the binding of FasL or TRAIL to the death receptor Fas or TRAILR induces the formation of DISC, activates caspase-8, and executes apoptosis^82^. However, in the absence of cIAPs or inhibition of caspase-8, RIPK1 translocates to the membrane^93^ and promotes the formation of complex II-b^83^ and initiation of necroptosis when Fas/TRAILR is activated ^78,79,84,85^.

Activation of TLRs induces the formation of a platform that recruits the cytoplasmic adaptor protein TRIF (Toll/IL-1 receptor domain-containing adaptor protein inducing interferon-Β). TRIF is involved in the activation of NF-κB signaling and induction of type I IFNs^86^. By relying on its RHIM (RIP homotypic interaction motif) domain, TRIF can interact with RIPK1 and RIPK3. In the presence of the apoptosis inhibitor zVAD-fmk, activation of TLR4 by lipopolysaccharide or activation of TLR3 by polyinosine–polycytidylic acid can induce TRIF-mediated necroptosis^87, 88^, which can be blocked by the inhibition of necrostatin-1 (Nec1) or knockout of RIPK1^89^. These results suggest that the RIPK1-TRIF signaling complex plays an important role in TLR3/4-induced necroptosis. In the absence of RIPK1, TRIF can also induce necroptosis by directly recruiting and activating RIPK3^87,89^.

**Fig. 4:**
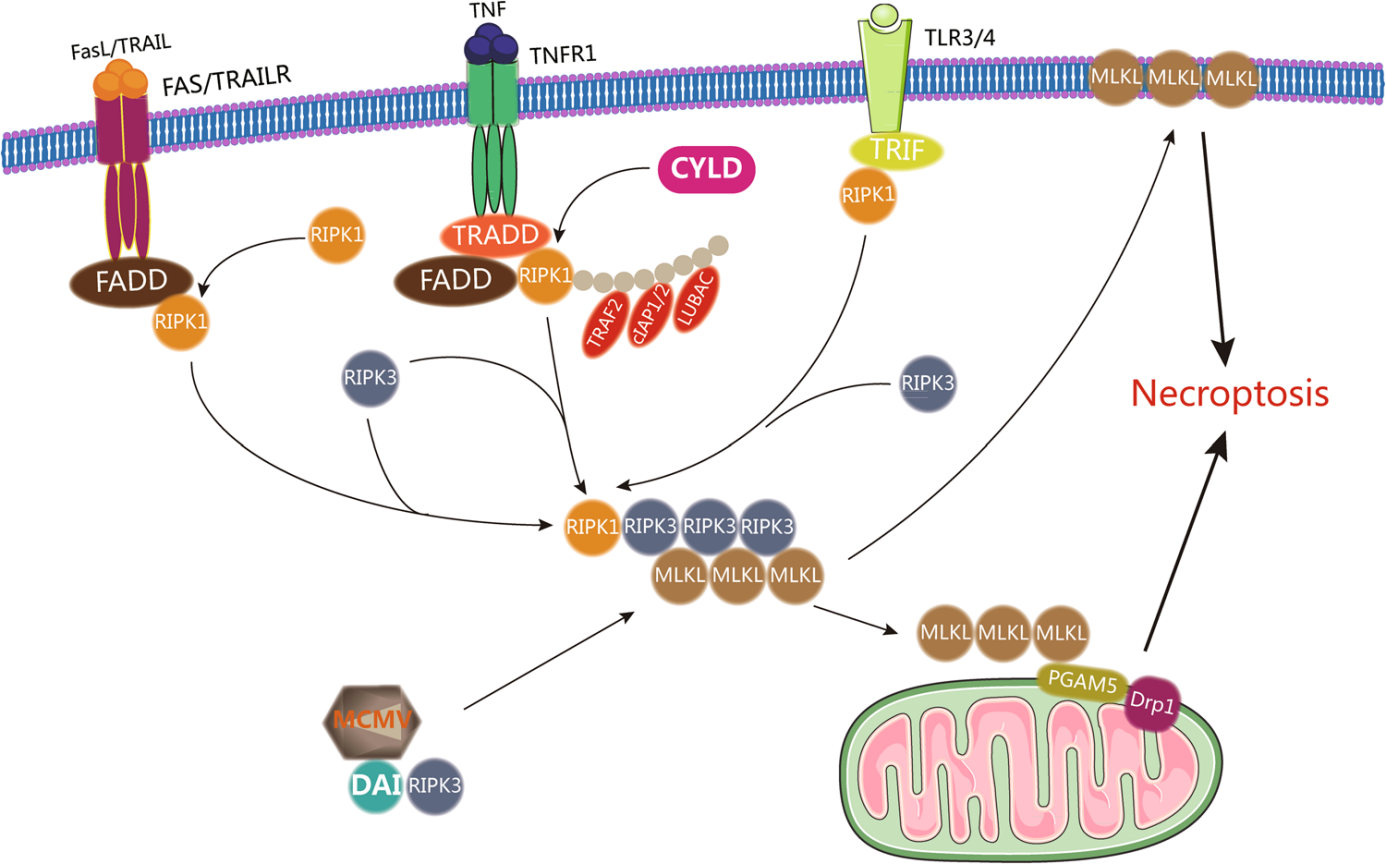
Mechanisms of necroptosis. In TNFR signaling, complex I containing TRADD, RIPK1, TRAF2, E3 ubiquitin ligases, cIAP1/2, and LUBAC is unstable when RIP1K is deubiquitinated by CYLD, leading to the formation of the necrosome together with high levels of RIPK3 and MLKL as well as inhibited caspase-8. Subsequently, RIPK3 in the necrosome oligomerizes and is phosphorylated, leading to the recruitment and phosphorylation of MLKL, and phosphorylated MLKL translocates to the plasma membrane to cause membrane damage and necroptosis, or phosphorylated MLKL interacts with phosphorylase PGAM5 on the mitochondrial membrane and then activates mitochondrial fission factor Drp1 to induce necroptosis. In Fas/TRAILR signaling, when cIAPs are absent and caspase-8 is inhibited, the activation of Fas/TRAILR can induce necroptosis. In TLR3/4 signaling, their activation can induce TRIF-mediated necroptosis in the presence of zVAD-fmk, TLR4 activation by lipopolysaccharide (LPS) or TLR3 activation by polyinosine–polycytidylic acid. In DAI signaling, in response to viral double-stranded DNA, DAI also mediates RIPK3-dependent necroptosis under certain conditions

In addition to death receptor signaling and TLR signaling, DNA-dependent activator of IFN-regulatory factors (DAI), a cytoplasmic viral DNA sensor^90,91^, can also induce necroptosis. Like TRIF, DAI has the RHIM structure. In response to viral (such as murine cytomegalovirus) double-stranded DNA, DAI can activate NF-κB, induce type I IFNs, and mediate RIPK3-dependent necroptosis^91^. Moreover, Wei et al. recently reported a novel necrosis mechanism, indicating that the acute cell necrosis induced by cationic nanocarriers occurs through the impairment of Na^+^/K^+^-ATPase, which causes a subsequent inflammatory response^92^. A diagram of the process of classical necroptosis is shown in Fig. 4.

## Associations among the three types of cell death

### Association between apoptosis and necroptosis

Apoptosis and necroptosis may occur simultaneously^93^ or mutually transform because of the interconnection of the downstream death signaling pathways. For example, cells can commit to necrotic cell death when apoptosis is blocked^94^, and oxidative stress-induced necrotic cell death involves the activation of the apoptosis-associated caspase-8/Bid pathway^95^. The final form of cell death will depend on the cell type, cell microenvironment, and initial inducers.

#### Caspase-8 and RIPK1/3

As an apoptotic initiator caspase, caspase-8 interacts with FADD to form DISC, followed by homologous dimerization and proteolytic processing. Activated caspase-8 is then released from DISC and triggers downstream apoptotic signaling^96^. Meanwhile, caspase-8 can inhibit necroptosis by cleaving and inactivating RIPK1 and RIPK3^85,97,98^. However, when the activity of caspase-8 is inhibited pharmacologically, such as by the pan-caspase inhibitor ZVAD, or genetically, such as in RNAi-mediated knockdown, RIPK1 and RIPK3 become activated through phosphorylation and induce the formation of the necrosome to trigger necroptosis^12,99,100^.

#### ATP

ATP plays a crucial role in the decision of cell death fate^101^. A high level of intracellular ATP often favors apoptosis, whereas a low level often promotes necrosis^102^. Therefore, excessive consumption of intracellular ATP or the inhibition of ATP synthesis may convert apoptosis to necrosis^101,103^. For example, substantial DNA damage leads to the activation of poly ADP-ribose polymerase-1 (PARP-1), a nuclear enzyme involved in DNA repair, resulting in the consumption of many NAD+ and ATP molecules and subsequent necrotic death^104,105^. The mitochondria is the major site that generates ATP; therefore, mitochondria dysfunction can trigger necrosis by ATP depletion. In addition, excessive mitochondrial ROS formation and the onset of the mitochondrial permeability transition are also causally linked to the conversion of apoptosis to necroptosis^106^.

### Association between autophagy and apoptosis

Autophagy is an intracellular catabolic mechanism that involves the degradation and recycling of cytoplasmic undesired components, such as malfunctioning proteins or damaged organelles, to maintain cellular homeostasis^107–109^. Autophagy is a double-edged sword and can either protect cells from apoptosis^110^ or promote apoptosis^111^ depending on the cell type, intracellular metabolic activity, extracellular nutrient supply and triggering stimuli.

#### Beclin1

Mammalian Beclin1 (Atg6 in yeast) cross-regulates autophagy and apoptosis through direct interaction with anti-apoptosis family members^112–115^. Beclin1 is a key molecule involved in the autophagosome formation. Beclin1 interacts with class III type PI3KC3/Vps34 and promotes the formation of the Beclin1-Vps34-Vps15 core complex^116,117^. Beclin1 is also a member of the BH3-only protein family. The antiapoptotic protein Bcl-2 or Bcl-xL combines with Beclin1 through the BH3 domains^118^ to simultaneously block the process of autophagy by inhibiting Beclin1 activity^119^ and the occurrence of endogenous apoptosis^111,118,120,121^. On the other hand, NOXA and other BH3-only family proteins can displace Bcl-2 family members from Beclin1 and promote autophagic cell death^122,123^. Furthermore, Beclin1 can also be cleaved by several caspase proteins, such as caspase-8 and caspase-3, to shift the cell fate from autophagy to apoptosis^14, 93^. The C-terminal fragment of Beclin1 can then translocate to the mitochondria and induce mitochondrial membrane permeability and apoptosis^124^.

### Association between autophagy and necroptosis

The interconnection between autophagy and necroptosis has been investigated in several studies with conflicting results. As a protective mechanism, autophagy can unsurprisingly inhibit necroptosis^125,126^. Interestingly, autophagy appears to facilitate necroptosis in certain instances^127^. Khan et al.^128^ reported that palmitic acid triggers Ca2+-dependent autophagy, resulting in the necroptosis of endothelial cells.

#### mTORC1

mTORC1 is a key sensor of nutrients, growth factors, and stress and controls cell metabolism, growth, and survival. Activation of mTORC1 by growth factors and nutrients can suppress autophagy by phosphorylation of autophagy-related proteins involved in autophagy initiation, such as ULK1 and ATG13, in the ULK complex and ATG14 in the VPS34 complex^129^. Cellular metabolic and energetic status regulates mTORC1 activity and consequently impacts autophagy induction. At a low-energy status, AMPK signaling is activated in response to an increased AMP/ATP ratio, which inhibits the mTORC1 signaling pathway via the phosphorylation of TSC2 (tuberous sclerosis complex 2), a mTORC1 negative regulator^13,41,130–133^, subsequently promoting autophagy^141,130,134^. Autophagy stimulation by the downregulation of mTORC1 signaling protect cells from programmed cell death, including apoptosis and necroptosis, under nutrient- or energy-deprived conditions^135–142^. As mentioned above, the activation of PARP-1 in the DNA damage response causes necrosis owing to ATP depletion, which also leads to AMPK activation, mTORC1 inhibition, and autophagy induction as the last protective resort^143^. The balance between autophagy and necrosis will determine the cell death fate.

### Association among apoptosis, autophagy, and necroptosis

#### Cellular FILCE-like inhibitory protein (cFLIP)

FADD-like interleukin-1β-converting enzyme (FLICE)-like inhibitory proteins (FLIPs) possess caspase-8-like structures but lack proteolytic activity. cFLIP has three major isoforms in humans containing one long protein cFLIP_L_ and two short proteins cFLIPs and cFLIP_R_^144–146^. cFLIP_L_ possesses a C-terminal caspase-8-like domain but does not have enzymatic activity due to the substitution of several catalytically important amino-acid residues^144–147^, whereas the other isoforms cFLIPs and cFLIP_R_ do not possess the caspase-like C-terminal domain. Nevertheless, these three isoforms all contain two death receptor domains at the N-termini, which allow them to interact with the adaptor protein FADD to form the DISC complex.

cFLIP regulates not only the death receptor-mediated extrinsic apoptosis pathway but also death receptor-independent apoptosis pathways. In complex IIb/ripoptosome, homodimeric caspase-8 initiates apoptosis by cleaving RIPK1 and disassembling complex IIb/ripoptosome. When procaspase-8 forms a heterodimer with cFLIP_L_, not only is necroptosis prevented due to cleavage of RIPK1 but apoptosis is also blocked because activated caspase-8 is not formed^148^. However, formation of a heterodimer by procaspase-8 with cFLIP_S/R_ triggers necroptosis owing to the lack of proteolytic cleavage of RIPK1^149–151^ and simultaneously fails to induce caspase-8-dependent apoptosis. Therefore, the existence of cFLIP isoforms in the ripoptosome determines whether cells will execute RIPK1-dependent necroptosis or caspase-8-dependent apoptosis.

**Fig. 5:**
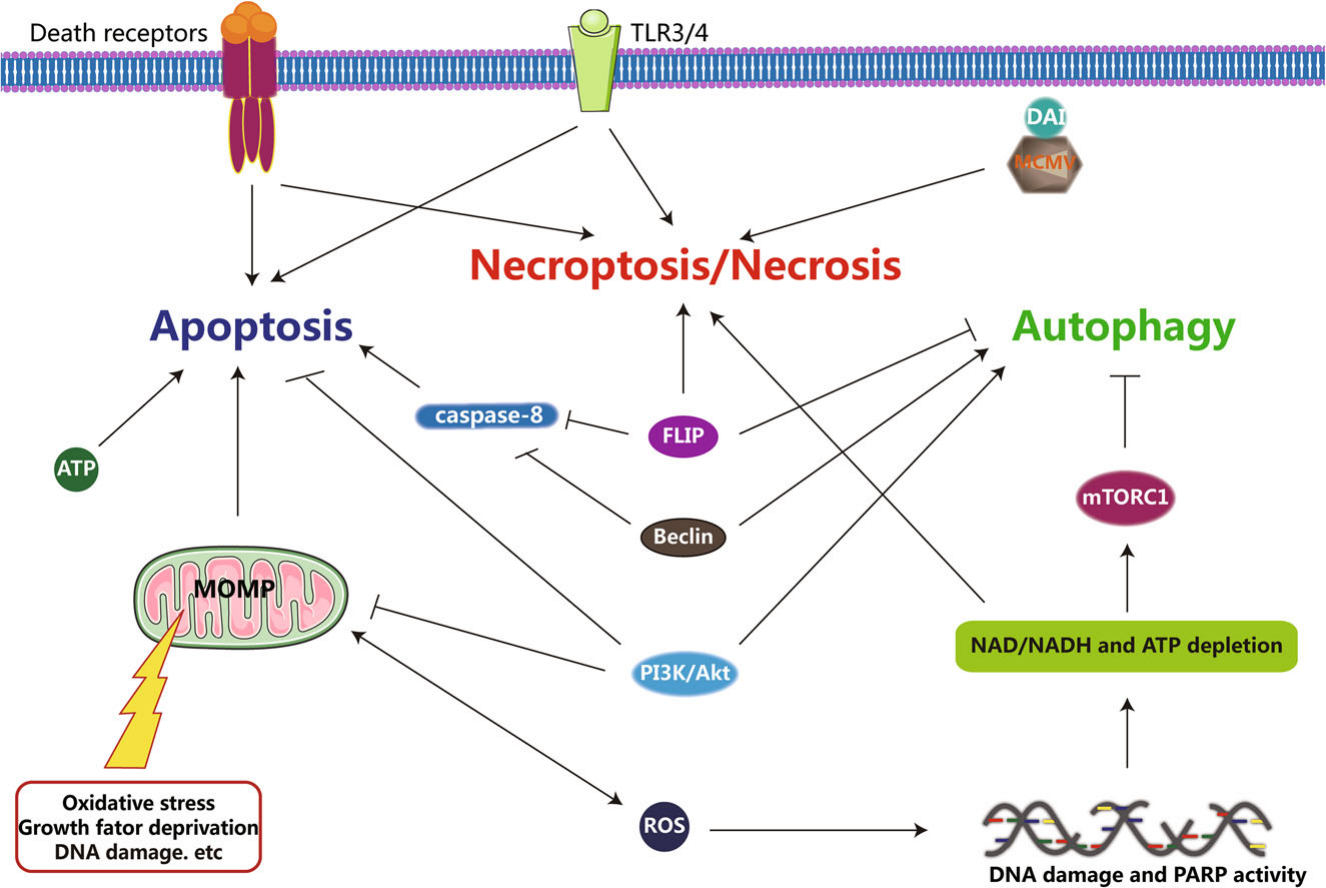
The relationship among the three types of cell death. FLIP regulates the modes of cell death by interacting with caspase-8, interfering with the functions of RIPK1, and combining Atg3 and LC3 by competitively binding Atg3. Caspase-8, which is a key factor in apoptosis, inhibits necroptosis by hydrolyzing RIPK1 and RIPK3. The level of intracellular ATP plays a crucial role in the decision of cell fate between apoptosis and necrosis. High levels of intracellular ATP often favor apoptosis, whereas low levels of intracellular ATP often favor necrosis. Beclin1, which is a key molecule required for autophagosome formation, can control the switch between autophagy and apoptosis via several mechanisms, such as by combining with Bcl-2 or Bcl-XL, which are anti-apoptotic proteins, and becoming hydrolyzed by several caspase proteins. mTOR can sense the level of intracellular ATP and relieve the inhibition of autophagy when the level of intracellular ATP is low, triggering necrotic cell death. When activated by growth factor, AKT can induce mTOR signaling to inhibit autophagy. The activation of AKT can inhibit apoptosis by phosphorylating apoptotic factors, such as Bad and caspase-9

In addition to the regulation of apoptosis and necroptosis, cFLIP is known to be a negative regulator of autophagy. During autophagosome formation, Atg3 covalently binds the microtubule-associated protein LC3. Strikingly, cFLIP prevents the combination of Atg3 and LC3 by competitively binding Atg3 and consequently inhibiting autophagy^152^. The process in which cFLIP regulates apoptosis and necroptosis through formation of the ripoptosome occurs at the plasma membrane, but cFLIP inhibits autophagy at the sites where autophagosomes form. Thus, the different subcellular localizations of cFLIP may be important for its actions^152^. The complex associations among the three types of cell death are summarized in Fig. 5.

## Cell death and disease treatment

### Cell death in neurodegenerative diseases

Neurodegenerative diseases, including Alzheimer’s disease, Parkinson’s disease, Huntington’s disease, and amyotrophic lateral sclerosis^153^, involve the loss of structures and functions of nerve cells owing to the accumulation of abnormal proteins in the intracellular and extracellular spaces^154^. Deregulated cell death has been implicated as a major mechanism in these neurodegenerative diseases. The intervention of cell death pathways is therefore considered a potential therapeutic strategy.

The interaction between β-amyloid accumulation and the death of neurocytes contributes to the progression of Alzheimer’s disease^155^. β-amyloid accumulates in mitochondria, mediates mitochondrial toxicity, and induces caspase-3-dependent apoptosis^156^, which, in turn, accelerates the formation of β-amyloid and neurofibrillary tangles^157^. Activation of caspases also cleaves autophagy-related proteins, such as Beclin1, consequently inhibiting autophagy^158^.

An overwhelming body of evidence indicates that the inhibition of programmed cell death may be an effective strategy for the treatment of neurodegenerative diseases. For example, the efficacy of minocycline in the treatment of Alzheimer’s disease and Parkinson’s disease has been linked to its anti-apoptosis action by inhibiting oxidative stress, cytochrome c release, and caspase-3 activation and by increasing the expression levels of anti-apoptotic proteins, such as Bcl-2^159^. Blocking necroptosis by the RIPK1 inhibitor Nec-1 protects cortical neuronal cells of embryonic rats and mouse hippocampal neurons from excitatory toxicosis-induced cell death^160,161^. Nec-1 can also reduce the death of striatal neurons with Huntingtin (HTT) mutations and retard the process of Huntington’s disease in HTT-mutant transgenic mice^162^. As a cell survival mechanism, promoting autophagy^163,164^ by mTOR inhibitors, such as rapamycin and its analogs (CCI-779, RAD001, and AP23573), protects cells from apoptotic and necrotic cell death^165,166^ and has been proven to be effective in the treatment of neurodegenerative diseases.

### Cell death in cancer

A delicate balance between cell death and survival maintains the homeostasis within a cell, a tissue, and an organism. Uncontrolled cell proliferation and escape from cell death have been recognized as the hallmarks of cancer cells^167^.

Substantial studies have suggested that autophagy plays a dual role in tumorigenesis. At the early stage, autophagy exerts an antitumor effect and curbs chronic tissue damage, inflammation, and genome instability; however, during the late stage, autophagy meets the energy and nutrient requirements to sustain tumor development^168^. Therefore, autophagy inhibitors, such as chloroquine or hydroxychloroquine, have a therapeutic potential in cancer treatment^169–171^. Autophagy also contributes to the resistance to cancer therapy. For example, autophagy plays a protective role against quercetin or histone deacetylase inhibitor SAHA (suberoylanilide hydroxamic acid)-induced apoptosis, and the combination of autophagy inhibitors and quercetin or SAHA may provide a rational utility of these drugs in the clinic^172,173^. Similarly, inhibition of autophagy has been reported to enhance the sensitivity of tumor cells to TRAIL agonists and promote apoptosis^170^.

Cancer cells exhibit aberrant apoptotic signaling, including upregulation of anti-apoptotic molecules and suppression of proapoptotic molecules^174–176^. The high expression level of survivin, a member of the IAP family^177^, the disrupted balance among the Bcl-2 family members^178^, or the impaired activity of caspases^179^, enhances cancer cell survival and is associated with tumor aggressiveness and the survival of cancer patients. Therefore, targeting apoptosis-related proteins has been one of the most active research fields in cancer therapeutics for the long-term and has achieved significant progress recently. The BH3 analog ABT-737 and its derivative ABT-263 act as pan-Bcl-2 inhibitors and simultaneously inhibit several members of Bcl-2 family proteins, including Bcl-2, Bcl-xL, and Bcl-W, to induce cell apoptosis^180,181^. BH3 analogs have shown therapeutic benefits for solid tumors and hematological malignancies^182,183^. The negative regulatory role of cFLIP in apoptosis makes it an attractive target molecule to treat cancer. The modulation of cFLIP expression and activity has been linked to the antitumor action of several targeted therapies, including those utilizing mTOR inhibitors and histone deacetylase inhibitors^184–186^.

Cell death-related pathways participate in the cellular stress response. Cancer cells exposed to various stresses (for example, DNA damages, oxidative stress) during oncogenic transformation and adaption to these stresses are required for cancer cells to survive. Oxidative stress results from the accumulation of ROS. ROS collectively include superoxide onion, hydrogen peroxide, and hydroxyl radical and regulate programmed cell death. Activation of death receptor-mediated signaling pathways has been associated with ROS production^187–190^. Wang et al.^191^ demonstrated ROS-mediated degradation of cFLIP, a negative regulator of Fas-induced apoptosis in lung epithelial cells. ROS can also trigger the intrinsic apoptotic cascade by disruption of the mitochondrial membrane potential, promoting cytochrome c detachment from cardiolipin and release to the cytosol and inducing oxidative mitochondrial DNA damage^192^. Furthermore, ROS can activate apoptotic signaling via ASK1/JNK signaling^193^. Accumulation of ROS can also activate autophagy. ROS-mediated oxidative modulation of Atg4 inhibited Atg4-delipidating activity, leading to the accumulation of LC3-PE on autophagosomal membranes and facilitating autophagosome formation. The interplay between ROS and autophagy contributes to cancer progression^194^. In the early stage of cancer initiation, autophagy is proposed to play a tumor suppressor role by reducing ROS accumulation via the degradation of ROS-producing mitochondria, thus limiting genomic instability^195^. However, at the later stage of tumor progression, autophagy may be exploited by cancer cells to promote their survival and oncogenic mutations^196^. ROS-mediated programmed cell death makes ROS-based therapies an attractive strategy in cancer treatment. Moreover, although cancer cells have developed redox adaptation mechanisms to survive in a high oxidative environment, extensive studies have suggested that they are more vulnerable to oxidative stress caused by ROS-generating agents than normal cells, providing the selectivity of ROS-based therapies^197^. Exogenous ROS-generating agents used as a single agent or in combination with other standard therapies have shown promise in pre-clinical studies^198–200^.

### Cell death in other diseases

In addition to neurodegenerative diseases and cancer, cell death is associated with other diseases. Autophagy is involved in intestinal homeostasis^201^, muscular dystrophy, stroke, pancreatitis, heart disease, liver disease, and type II diabetes^202–205^. Increasing the activity of Beclin1 has been proposed as a therapeutic strategy for these autophagy-related diseases^202^. Apoptosis is also associated with ischemic stroke, acute central nervous system injury, heart disease, infectious diseases, autoimmune diseases^29^. Therefore, targeting apoptotic pathways such as the broad-spectrum caspase inhibitor Q-VD-OPh, has been reported to cure diseases induced by apoptosis^206–209^. Necroptosis has been linked to the pathogenesis of ischemia-reperfusion injury^210^, multiple sclerosis^211^, myocardial infarction, stroke^63,212^, inflammatory disease^213^, acute kidney injury^214^, and microbial infection^184,187,215–217^. N^e^c-1, an inhibitor of RIPK1, has shown the efficacy by preventing necroptosis^64,164,212,214,218–220^.

## Prospects

Apoptosis, autophagy, and necrosis, three types of cell death, have been studied separately and are considered independent processes. Recent advances in cell death research have changed our perception, leading us to consider these processes as interconnected with overlapping signaling pathways and cross-talk in response to different stresses. The existence of diverse regulated cell death pathways implicates the complexity of cell death programs but also provides novel therapeutic targets. Further studies are required to investigate the linkage within different cell death programs and identify key molecular factors that determine cell death under specific pathological conditions and that can be pharmacologically manipulated.
